# Enabling reproducible re-analysis of single-cell data

**DOI:** 10.1186/s13059-021-02422-y

**Published:** 2021-07-26

**Authors:** Michael A. Skinnider, Jordan W. Squair, Grégoire Courtine

**Affiliations:** 1 Brain Mind Institute, Faculty of Life Sciences, École Polytechnique Fédérale de Lausanne (EPFL), Lausanne, Switzerland; 2 Michael Smith Laboratories, University of British Columbia, Vancouver, British Columbia Canada; 3 NeuroRestore Center, Department of Clinical Neuroscience, Lausanne University Hospital (CHUV) and University of Lausanne (UNIL), Lausanne, Switzerland; 4 Center for Neuroprosthetics, Faculty of Life Sciences, École Polytechnique Fédérale de Lausanne (EPFL), Lausanne, Switzerland

The maturation of single-cell technologies is transforming our understanding of health and disease. Reflecting this promise, the number of studies reporting single-cell analyses has grown exponentially over the past decade [1]. The vast majority of the raw sequencing data generated by these studies are deposited in public repositories, reflecting strong expectations on data availability enforced by the community, funding agencies, and journals. However, similar standards for the deposition of processed data are still in their infancy [2]. Here, we report on the availability of processed datasets accompanying published single-cell transcriptomics studies. We attempted to re-analyze 72 published scRNA-seq datasets but found that only 35 (49%) could be fully reconstructed from publicly available data. Whereas both the raw sequencing reads and processed gene expression matrices were almost always available, the cell types inferred from single-cell gene expression profiles often were not. Our findings highlight the widespread omission of metadata required to reproduce and extend published analyses.

Explosive growth in single-cell genomics has spurred investigators to generate hundreds of datasets. This wealth of published data provides an unprecedented resource that can be used to address many new biological questions. For instance, single-cell RNA-seq (scRNA-seq) data have been integrated with genome-wide association study (GWAS) results to identify cell types underlying complex traits [3, 4]. Publicly available single-cell datasets also provide a fertile ground to evaluate new computational methods for single-cell data [5] and a basis to assemble comprehensive cell atlases through data integration efforts [6].

The need to provide both raw and processed functional genomics data in a standardized format has long been recognized. A minimum information standard was proposed for microarray data in 2001 (MIAME [7]) and subsequently updated for high-throughput sequencing (MINSEQE). However, single-cell technologies differ in important ways from conventional, “bulk” assays with respect to data reporting. One particularly significant difference is that complete metadata at the level of samples is not sufficient to reproduce analyses at the level of individual cells.

An especially important aspect of cell-level metadata is the cell type inferred by investigators for each individual cell. The assignment of cell types from single-cell gene expression profiles is a laborious process that often involves multiple iterative rounds of clustering, sub-clustering, and cluster merging. This practice relies on extensive manual intervention by domain experts, who must negotiate between the results suggested by unsupervised clustering of high-dimensional data, and a broader body of knowledge about the biological system of interest. Despite efforts to automate this process [8, 9], these manually assigned cell type labels remain, for most purposes, the gold standard [10]. Consequently, the absence of cell type annotations from deposited data hinders the re-use of these datasets to address new biological questions. Moreover, the subjectivity inherent in cell type annotation implies that without the annotations established by the original authors, future investigators will be unable to reproduce published analyses.

A recent set of guidelines for reporting of single-cell experiments recognized the importance of cell type annotations [2]. However, these annotations were not included among the mandatory entries for a single-cell dataset (significance level 1); instead, they were deemed to “greatly improve data utility” (significance level 2). Here, we demonstrate that the frequent absence of cell type annotations impedes the re-analysis of many published datasets and prevents the reproduction of published findings. Consequently, we argue that this critical piece of metadata should be required for public data deposition.

We came face-to-face with this problem in our efforts to develop computational methods for comparative analysis of single-cell data [11, 12]. We sought to establish a large compendium of published single-cell datasets that would allow us to benchmark our methods against existing tools. To establish this compendium, we attempted to re-analyze a total of 72 published datasets (Additional file 1: Table S1). For each dataset, we attempted to locate four pieces of information required to reproduce and extend the published analysis, including (i) the processed gene expression matrix; (ii) the experimental condition and (iii) biological replicate from which each cell barcode was obtained (sample-level metadata); and (iv) the cell type assigned to each cell barcode (cell-level metadata). We searched extensively for this information across multiple public sources, including standard repositories such as the Gene Expression Omnibus, the supplementary information accompanying the publication, GitHub repositories containing published code, and study-specific websites, among other sources. In cases where one or more pieces of information were not publicly available, we contacted the corresponding authors to request the relevant data.

For the vast majority of the studies, both the raw sequencing reads (69/72, 96%; Additional file 2: Figure S1a) and the processed gene expression matrix (68/72, 94%; Fig. 1a and Additional file 2: Figure S1b) were publicly available. Both could generally be obtained from standard repositories, although a few datasets were distributed through the supplementary information accompanying the publication, or study-specific websites (Additional file 2: Figure S1c-d). Sample-level metadata, such as experimental condition (Fig. 1b and Additional file 2: Figure S1e) or biological replicate (Additional file 2: Figure S1f), was typically provided, although it was unavailable for a non-negligible number of studies (12/72, 17%). In marked contrast, nearly half of the studies failed to include any cell type annotations (31/69, 45%, excluding three studies in cell lines; Fig. 1c). Moreover, when present, these annotations often did not accompany the structured deposition. Instead, they were obtained from a more scattered range of sources and occasionally required substantial manual intervention to reconstruct (Fig. 1d).

**Fig. 1:**
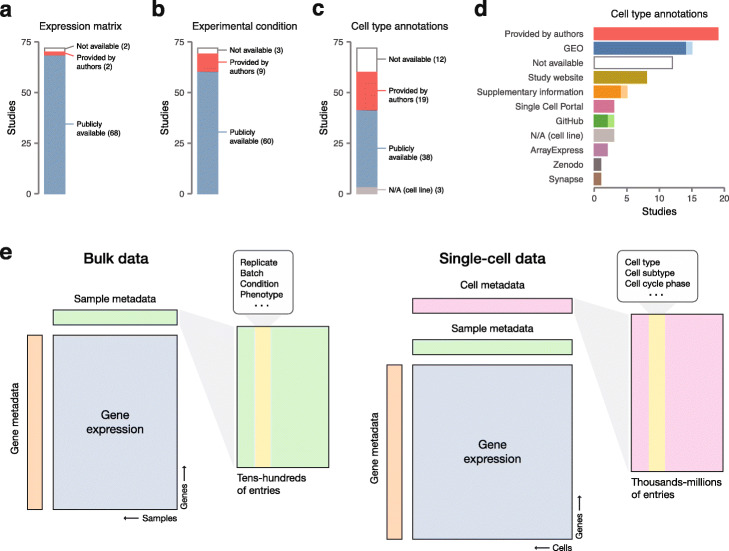
Availability of gene expression data and metadata for 72 published scRNA-seq datasets. **a** Availability of processed gene expression matrices. **b** Availability of sample-level metadata, as exemplified by experimental condition (e.g., treatment vs. control). **c** Availability of cell-level metadata, as exemplified by cell type annotations for each cell barcode. **d** Sources from which cell type annotations were obtained. Light shades represent datasets for which two or more additional manual processing steps were required to obtain cell types. **e** Schematic overview of the differences in data reporting required for bulk vs. single-cell assays. Processed single-cell data is characterized by an additional level of cell-level metadata not captured by the sample-level metadata required for deposition of bulk datasets

In total, we were able to completely reconstruct only 35 of 72 datasets (49%) from publicly available information. To obtain the relevant data from the remaining 37 datasets, we contacted the authors of the corresponding studies. These efforts led us to reconstruct 18 additional datasets (for a total of 74%; Fig. 1a–c).

Our efforts establish that a considerable fraction of the data required to reproduce or extend published analyses is not publicly available from any source. That a large fraction of the unavailable data could be obtained by corresponding with the authors suggests that this situation does not primarily reflect a desire on the part of investigators to keep their data private. Instead, we conclude that the primary issue is a lack of standards for the deposition of cell-level metadata, which has not been relevant to previous guidelines developed for “bulk” data (Fig. 1e). Although guidelines have recently been proposed for single-cell data deposition [2], these guidelines have primarily focused on describing experimental aspects of the study and suggest that cell-level metadata is not required to provide a complete description of the data. While these guidelines provide an important step towards improving the reusability of deposited data, we argue that metadata deposited to public repositories must include the cell types assigned by investigators for each cellular barcode. Otherwise, the field risks a scenario in which many published analyses cannot be reproduced, and much of the data deposited to public databases cannot be productively re-used without substantial computational proficiency and domain expertise.

A secondary issue is that no mechanism currently exists to enforce the format in which processed single-cell datasets are provided. In the absence of a uniform format for processed data deposition, we suggest that the responsibility for ensuring the availability of all necessary metadata falls on the shoulders of peer reviewers. Consequently, a recommendation by journals that reviewers inspect deposited data for the presence of appropriate cell-level metadata has the potential to significantly improve reproducibility.

Although the availability of cell type annotations is critical to reproduce published analyses, investigators must be careful not to take these annotations as the ground truth. The manual and subjective process by which cell types are annotated and the limitations of existing computational tools both have the potential to introduce error into published annotations. Moreover, for some forms of re-analysis, investigators may find it beneficial to update cell type annotations from scratch, either from a count matrix or the raw reads themselves [13, 14]. However, even in scenarios where error is suspected or cell type annotations are to be updated, the availability of the original cell type annotations provides a valuable basis for comparison.

In view of the effort required to reconstruct this corpus of data, we provide all 35 publicly available datasets (Additional file 2: Figure S2) that we curated and assembled in analysis-ready formats to facilitate method development or secondary analyses (available from Zenodo: 10.5281/zenodo.4772064). All code used to download and preprocess these datasets is available from GitHub (https://github.com/neurorestore/single-cell-repository).

## Supplementary Information

**Additional file 1** Table S1.

**Additional file 2** Figures S1-S2.

## Data Availability

Processed publicly available datasets are available from Zenodo (10.5281/zenodo.4772064). Source code used to download and preprocess all raw data from publicly available repositories, including all relevant accession codes, are available from GitHub (https://github.com/neurorestore/single-cell-repository) under the MIT license.
