# Supplementary material for: Enabling reproducible re-analysis of single-cell data
Source: Genome Biol. 2021 Jul 26;22:215. doi: 10.1186/s13059-021-02422-y (PMC8311938; doi:10.1186/s13059-021-02422-y)
Supplement: Supplementary file 2 — Additional file 2 Figures S1-S2. [file 13059_2021_2422_MOESM2_ESM.pdf]

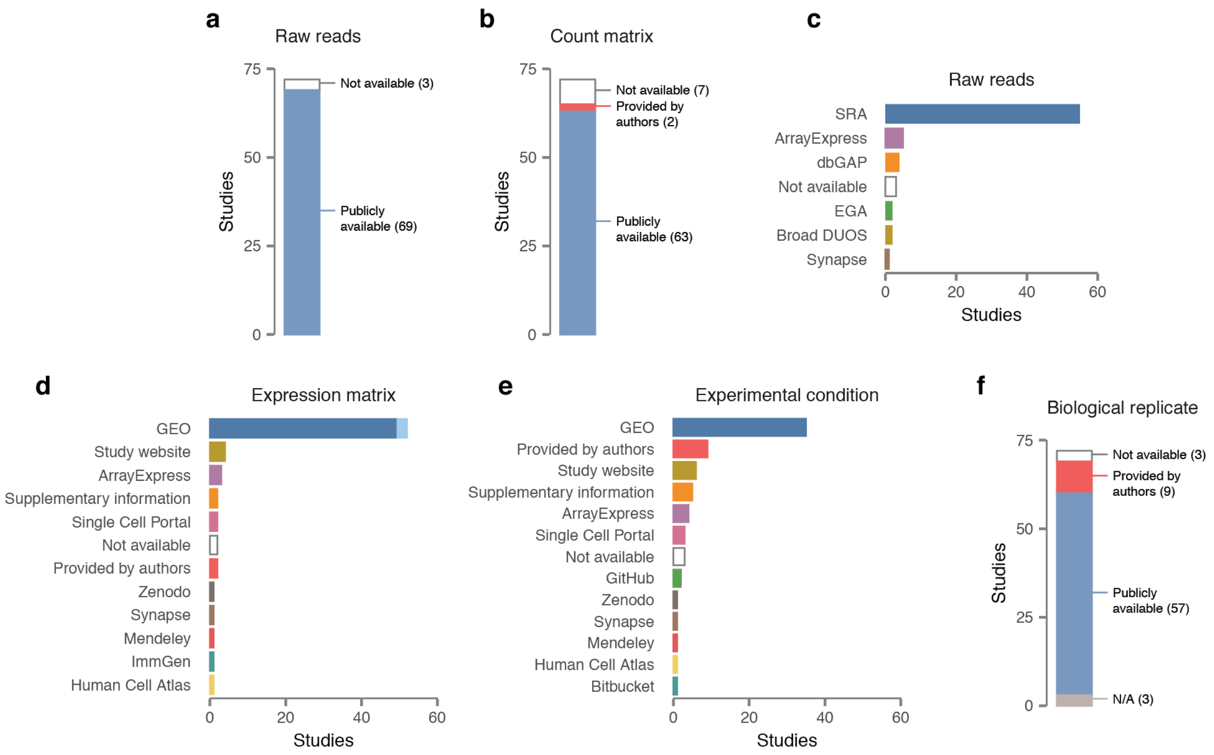

Supplementary Fig. 1 | Availability of additional gene expression data and metadata for 72 published scRNA-seq datasets.
a, Availability of raw sequencing reads.
b, Availability of non-normalized gene expression count matrices.
c, Sources from which raw sequencing reads were obtained.
d, Sources from which gene expression matrices were obtained.
e, Sources from which experimental conditions for each cell were obtained.
f, Availability of sample-level metadata, as alternatively exemplified by biological replicate.

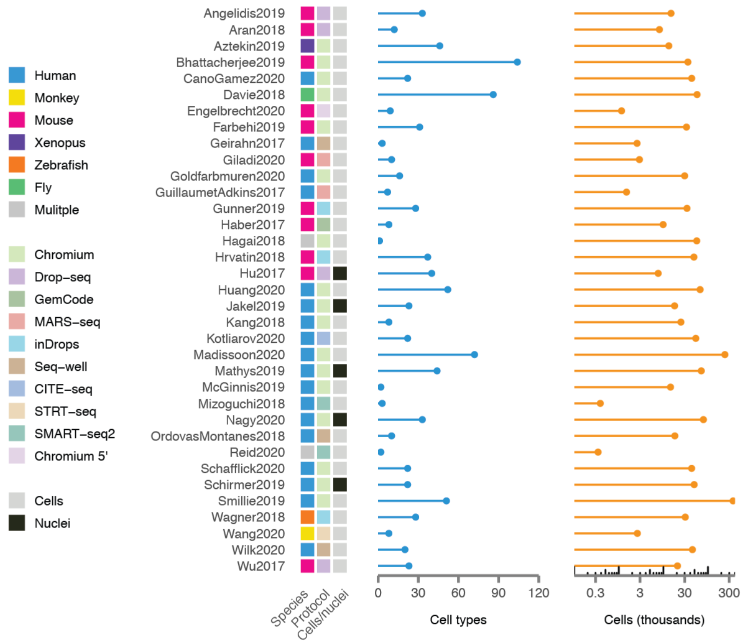

Supplementary Fig. 2 | Overview of 35 scRNA-seq datasets for which all data was publicly available.
